# PSYCHOSOCIAL WORKING CONDITIONS AND MENTAL WELL-BEING OF REMOTE AND STATIONARY EMPLOYEES: A LONGITUDINAL STUDY

**DOI:** 10.13075/ijomeh.1896.02526

**Published:** 2025

**Authors:** Dorota Żołnierczyk-Zreda, Łukasz Kapica, Andrzej Najmiec, Joanna Kamińska, Joanna Mazur-Różycka, Joanna Bugajska

**Affiliations:** Central Institute for Labor Protection – National Research Institute, Ergonomic Department, Warsaw, Poland

**Keywords:** remote work, mental well-being, stationary work, longitudinal study, psychosocial working conditions, work life quality

## Abstract

**Objectives:**

The aim of the article is to compare people working remotely and people working at the employer's premises in terms of psychosocial working conditions and mental well-being.

**Material and Methods:**

A longitudinal study conducted on a group of 494 people working remotely (N = 206) and those working stationary (N = 288) in 2021–2022 using the *Copenhagen Working Conditions Questionnaire*.

**Results:**

The results of analysis of variance in the mixed design showed that some psychosocial conditions of their work (e.g., emotional work demands, demands for hiding emotions, control at work, role conflict and trust in co-workers) were assessed better by remote workers than by people working stationary. Remote workers also had better mental well-being over time than those working at the employer's premises, especially in relation to psychological strain, depression and burnout.

**Conclusions:**

The results of this study therefore suggest that remote work may be a desirable form of work from the point of view of improving psychosocial working conditions and the mental well-being of employees, and thus increase the quality of life of working people.

## Highlights

- Remote work becomes increasingly popular in Poland compared to prepandemic period.
- Some psychosocial work aspects are better in remote than in stationary work.
- A better mental well-being in remote workers is observed over time.

## INTRODUCTION

Due to globalization, digitalization and technological advances, the world of work has changed radically over the past decades. Both the content of work and the way it is organized and performed have changed. Among the various types of flexible work organization, teleworking, or working from home, has emerged. The COVID-19 pandemic has radically accelerated its widespread adoption in Europe and worldwide. As recently as 2015, only 17% of European workers were engaged in some form of teleworking [1]. In Poland, teleworking was very limited at the time, but after the outbreak of the pandemic, i.e., between April and May 2020 and February and March 2021, almost 40% of Poles were working remotely [2]. Higher percentages were found, for example, in the Netherlands (59.6%), Belgium (59.1%), and Finland (58.6%).

The pandemic has also radically changed attitudes towards remote working among both employers and employees. According to a report by CIONET, Deloitte and VMware [3], as many as 88% of employers said they would remain with partial remote working after the pandemic had ended. Also, a study by Davis et al. [4] shows that after a pandemic, home offices are likely to be the workplace for a large part of the population, both in the short and long term. Another important aspect is that employers have realized that work can be done at home, which is likely to lead to regular working hours done at home in the long term. Many companies intend to maintain the possibility of remote working especially in a hybrid form (part of the time working remotely and part of the time at the employer's premises). They cite the benefits of this type of working for both the organization and the employees themselves, including: improved productivity, reduced home-work-home travel costs, savings in employee time and company organizational resources, higher job satisfaction.

Remote working is also very popular among employees themselves. After experiencing this form of work for some time, employees believe that this nature of work should be maintained to some extent in the future. In a short time, these needs have been reflected in Polish law, as the Remote Working Act [5] has been in force since April 2023. The Act provides for both total remote work and hybrid remote work (partly at home, partly at a company), according to the needs of the individual employee and the employer. Occasional remote work is granted at the request of the employee in an amount of 24 days/calendar year. Existing research on the impact of amendments to the Labour Code related to remote working indicates that 54% of hybrid workers and 50% of remote workers believe that this type of work will have a positive impact on their psychological wellbeing [6].

The benefits of remote working are confirmed by previous results of both Polish [7–9] and foreign studies [10,11]. In particular, the possibility of working flexible hours, spending less time commuting to work more free time, a sense of control, more time for family and friends, and choice about where to stay and work are highlighted in this context.

There is also evidence in the literature pointing to the negative consequences of working from home with regard to employees' psychological well-being [12–15]. Disadvantages of working remotely include less opportunity for promotion, social isolation, problems with work-home balance, compromised job security, lack of knowledge and experience sharing between employees, family-work conflict, and lack of suitable working conditions. Another indicated source of stress for people working remotely is work overload, often resulting from difficulties in organizing working time and work tasks, the need to quickly master other, previously unfamiliar computer programs, as well as previously unpracticed forms of contact with clients, patients and students for representatives of many service, educational and medical professions. What we do not know is how these demands affect worker autonomy, and which other aspects of the psychosocial demands of work become challenging for the worker.

The aim of the research reported in this article is to add to this knowledge by comparing many of the psychosocial demands of work and also the psychological wellbeing of those who work remotely from home and those who do their work on the employer's premises. In order to increase the validity of the results, the study was conducted in a longitudinal design, as advocated by the authors of existing research reviews on this topic [16,17].

## MATERIAL AND METHODS

### Study design

The research is conducted in a longitudinal research paradigm, with an interval of about 1 year between measurements. The results of cross-sectional surveys with measurements of psychosocial working conditions made at the same time may be distorted by the current situation, especially in the context of the severity of the epidemic, which affects social wellbeing, including that of the subjects. The analysis of quantitative research results with repeated measurements requires the use of appropriate statistical methods.

The survey was conducted on an Ariadna panel using the computer assisted web interview (CAWI) method. Each respondent was assigned a unique ID number so that they could be identified in the database for the second measurement. Respondents were assured full anonymity when completing the survey.

### Participants

To select participants for the study, the following criteria of working in remote mode were defined:

– working on the employer's instructions,
– working for a specified period of time determined by the employer's instructions or the duration of an epidemic condition,
– working outside the place of its permanent performance,
– using means of direct communication at a distance or concerning the performance of a manufacturing part.

The criteria of working stationary were the following:

– working at the employer's/company's headquarters,
– performing tasks on working days, typically in an 8-hour work schedule.

The first survey involved 1459 people aged 18–76 years (mean (M) ± standard deviation (SD) 41.84±11.40 years) from 16 provinces. The sample consisted of 740 women (50.7%) and 719 men (49.3%). There were 738 employees doing remote work and 721 employees doing stationary work.

The second measurement included 494 people between the ages of 20 and 76 (M±SD 41.84±11.40) from 16 voivodeships. The sample consisted of 236 women (47.8%) and 258 men (52.2%).

In the second measurement, those who did not change the location and mode of their work between measurements were 206 employees working remotely and 288 employees working in a stationary mode.

Table 1 shows the characteristics of the study group in terms of gender, age, marital status, education and place of residence of total respondents.

**Table 1.** Demographic characterist of the study sample, remote workers (N = 206) and stationary workers (N = 288), working in the offices, nationwide longitudinal study, Poland, 2019–2020

Variable	Participants (N = 494)	
	n	%
Gender		
female		
remote	101	49.0
stationary	135	46.9
all	236	47.8
male		
remote	105	51.0
stationary	153	53.1
all	258	52.2
Age		
18–29 years		
remote	40	19.4
stationary	42	14.6
all	82	16.6
30–40 years		
remote	69	33.5
stationary	106	36.8
all	175	35.4
41–50 years		
remote	58	28.2
stationary	57	19.8
all	115	23.3
51–65 years		
stationary	39	18.9
all	83	28.8
remote	122	24.7
Marital status		
single		
remote	66	32.0
stationary	71	24.7
all	137	27.7
married		
remote	140	68.0
stationary	217	75.3
all	357	72.3
Education		
elementary		
remote	6	2.9
stationary	11	3.8
all	17	3.4
secondary		
remote	33	16
stationary	52	18,1
all	85	17.2
post-secondary		
remote	20	9.7
stationary	30	10.4
all	50	10.1
higher		
remote	147	71.4
stationary	195	67.7
all	342	69.2
Place of residence		
country		
remote	27	13.1
stationary	49	17.0
all	76	15.4
town (≤20 000 inhabitant)		
remote	13	6.3
stationary	33	11.5
all	46	9.3
city		
20 000–100 000 inhabitants		
remote	43	20.9
stationary	67	23.3
all	110	22.3
100 001–500 000 inhabitants		
remote	54	26.2
stationary	76	26.4
all	130	26.3
>500 000 inhabitants		
remote	69	33.5
stationary	63	21.9
all	132	26.7

The distribution of demographic variables characterizing remote and stationary employees was compared. Differences in age distributions between the 2 groups related to work mode were verified using the χ^2^ test. The statistically significant result concerned the age of the respondents (χ^2^(3, 494) = 12.588, p < 0.01) and place of residence (χ^2^(4, 494) = 10.988, p < 0.05). It turned out that among remote workers there is a greater representation of younger people and those living in larger cities (>500 000 inhabitants).

Due to the fact that as many as 965 people were eliminated in the second round, analyzes were also carried out to check whether this group of people differed significantly in terms of demographic variables. For this purpose, the distribution of variables in the group that took part in the second measurement (N = 494) and the group that did not take part in it (N = 965) was compared using the χ2 test. The analysis showed sample invariance between measurements in terms of gender and size of place of residence. However, older people were excluded from the study.

### Survey questionnaire

The research questionnaire included questions on well-being (e.g., psychological and physical well-being and job satisfaction), and psychosocial aspects of their work, as well as their individual/demographic characteristics. Part I of the questionnaire contained typical demographic questions (e.g. age, gender, marital status, education, place of residence). Part II of the questionnaire measured variables comprising psychosocial working conditions and employee mental health. The *Copenhagen Psychosocial Questionnaire* (COPSOQ II) measures a wide range of various psychosocial factors of the work environment that have a confirmed impact on employees' mental well-being [18], in particular, variables such as:

– work-family conflict,
– family-work conflict,
– quantitative demands,
– sense of influence at work,
– pace of work,
– emotional demands,
– attachment to the workplace,
– support from colleagues and superiors,
– opportunities for development,
– meaning of work,
– sense of predictability,
– rewards,
– role clarity,
– job insecurity,
– trust in management,
– fairness, respect, and quality of leadership,
– job satisfaction,
– burnout,
– psychological stress,
– cognitive stress,
– depression.

### Statistical analysis

The aim of the statistical analyses was to assess the differences in the study variables between remote and sedentary workers and between the first (2021) and second measurements (2022). The results of the COPSOQ II were compared using a mixed-model ANCOVA test. Due to differences in age group sizes and place of residence, these variables were controlled as covariates. Bonferroni correction was applied in pairwise comparisons. A value of 0.05 was used as the significance level. Analyses were performed using the jamovi R package (v. 2.3.28), which is built on the R language.

## RESULTS

### Comparative analysis of psychosocial working conditions of employees employed in remote and stationary work systems in 2 measurements

Table 2 shows the means and standard deviations of all examined psychosocial variables in the group of people working remotely and stationary in 2 measurements.

**Table 2.** Means and standard deviations of all examined psychosocial variables in the group of people working remotely (N = 206) and stationary (N = 288) in 2 measurements, nationwide longitudinal study, Poland, 2019–2020

Variable	COPSOQ score^a^						F(1, 490)	η_p_^2^
	remote work			stationary work				
	n^b^	M	SD	n^b^	M	SD		
Quantitative demands							0, 747	0.000
measurement 1	206	39.05	19.90	288	37.33	19.70	0, 290	0.001
measurement 2	206	37.62	19.26	288	37.22	20.37	1, 067	0.000
Cognitive demands							1, 118	0.000
measurement 1	206	57.37	20.28	288	57.05	21.52	0, 055	0.000
measurement 2	206	56.58	21.52	288	58.09	19.52	0, 354	0.000
Emotional demands							3, 982*	0.008
measurement 1	206	46.30	21.13	288	48.98	20.54	5, 098*	0.010
measurement 2	206	43.69	18.20	288	49.24	20.87	0, 020	0.000
Hiding emotios							3, 654	0.007
measurement 1	206	48.83	23.79	288	53.07	22.04	11, 670***	0.023
measurement 2	206	47.89	21.75	288	55.38	22.09	1, 817	0.004
Work pace							0, 031	0.000
measurement 1	206	51.54	23.22	288	51.45	21.17	0, 032	0.000
measurement 2	206	50.65	21.41	288	51.01	21.30	0, 182	0.000
Control							0, 011	0.000
measurement 1	206	50.27	25.19	288	46.57	22.07	5, 609*	0.011
measurement 2	206	51.27	21.70	288	47.24	21.07	1, 505	0.003
Development opportunities							0, 272	0.001
measurement 1	206	60.68	21.71	288	60.53	21.23	0, 151	0.0001
measurement 2	206	60.34	20.15	288	59.12	21.35	1, 130	0.002
Work variety							0, 037	0.000
measurement 1	206	52.12	20.68	288	51.87	20.21	0, 067	0.000
measurement 2	206	53.03	21.70	288	52.82	20.43	0, 009	0.000
Meaning of work							2, 325	0.005
measurement 1	206	63.27	23.47	288	63.11	21.95	0, 213	0.000
measurement 2	206	60.88	21.93	288	63.51	22.07	0, 129	0.000
Attachement to work							0, 811	0.002
measurement 1	206	57.31	20.64	288	58.03	21.11	0, 009	0.000
measurement 2	190	49.47	22.83	279	48.76	13.93	89, 642***	0.154
Sense of predicability							0, 003	0.956
measurement 1	206	58.19	22.44	288	56.51	23.07	0, 790	0.002
measurement 2	206	57.16	22.57	288	55.64	23.42	1, 647	0.003
Rewards							0, 009	0.000
measurement 1	206	62.26	23.07	288	59.55	23.61	1, 880	0.004
measurement 2	206	61.04	22.07	288	58.51	23.73	0, 718	0.001
Role clarity							0, 507	0.001
measurement 1	206	70.67	22.07	288	69.88	20.92	0, 144	0.000
measurement 2	206	69.38	20.99	288	69.44	21.58	1, 416	0.003
Role conflict							2, 147	0.004
measurement 1	206	37.80	20.96	288	43.27	20.95	6, 100*	0.012
measurement 2	206	39.32	21.15	288	42.04	20.76	0, 009	0.000
Leadership quality							0, 006	0.000
measurement 1	206	57.01	25.47	288	56.14	26.68	0, 006	0.000
measurement 2	206	55.89	24.38	288	55.36	24.67	0, 718	0.001
Coworkers support							0, 020	0.000
measurement 1	187	50.38	24.06	278	52.35	22.95	1, 422	0.003
measurement 2	191	49.44	22.83	279	51.46	22.72	0, 011	0.000
Supervisors support							0, 198	0.000
measurement 1	206	53.84	25.18	288	55.27	23.75	0, 597	0.001
measurement 2	206	54.17	24.35	288	55.01	23.32	3, 562	0.007
Social climate							0, 737	0.002
measurement 1	191	61.69	25.65	281	64.13	24.47	1, 130	0.002
measurement 2	203	60.30	25.88	281	61.89	23.63	0, 036	0.000
Life-work conflict							0, 215	0.000
measurement 1	206	17.39	23.24	288	15.91	22.40	0, 936	0.002
measurement 2	206	18.35	23.58	288	16.25	22.22	0, 029	0.000
Work-life conflict							4, 587*	0.009
measurement 1	206	35.15	23.58	288	35.88	24.72	1, 790	0.004
measurement 2	206	31.16	23.38	288	35.82	24.08	0, 035	0.000
Job insecurity							0, 201	0.000
measurement 1	206	31.07	23.51	288	31.45	22.09	0, 004	0.000
measurement 2	206	30.98	22.41	288	30.08	21.40	0, 310	0.001
Job satisfaction							1, 042	0.002
measurement 1	202	56.38	21.94	283	57.89	23.32	0, 115	0.000
measurement 2	206	56.14	21.54	285	55.93	22.38	0, 313	0.001
Trust in coworkers							0, 212	0.000
measurement 1	206	62.30	19.59	288	58.71	20.93	5, 520*	0.011
measurement 2	206	62.62	20.02	288	58.16	20.54	1, 059	0.002
Trust in management							0, 709	0.001
measurement 1	206	62.04	20.97	288	59.70	20.73	2, 610	0.005
measurement 2	206	62.14	20.62	288	58.66	20.31	2, 495	0.005
Justice and respect							0, 007	0.000
measurement 1	206	60.38	21.55	288	57.14	22.50	2, 890	0.006
measurement 2	206	58.59	22.93	288	55.30	22.53	2, 292	0.005
Social equality							1, 820	0.004
measurement 1	206	64.23	21.26	288	62.28	20.96	2, 090	0.005
measurement 2	206	65.02	24.36	288	60.79	23.32	2, 152	0.005
Self-efficacy							0, 402	0.001
measurement 1	206	56.31	19.81	288	53.67	21.67	3, 340	0.007
measurement 2	206	56.75	21.42	288	53.64	20.12	0, 001	0.000
General health							0, 470	0.001
measurement 1	206	52.79	25.81	288	55.38	22.61	2, 720	0.006
measurement 2	206	51.09	24.98	288	53.04	22.71	2, 991	0.006
Sleep problems							2, 634	0.005
measurement 1	206	34.80	24.59	288	32.57	24.49	0, 063	0.000
measurement 2	206	30.46	24.58	288	31.27	24.10	0, 825	0.002
Burnout							4, 709*	0.010
measurement 1	206	42.14	23.77	288	41.04	23.64	0, 465	0.001
measurement 2	206	37.77	23.57	288	40.71	22.78	2, 198	0.004
Psychological strain							4, 916*	0.010
measurement 1	206	41.20	24.08	288	38.87	22.78	0, 015	0.000
measurement 2	206	37.38	24.50	288	39.19	23.24	0, 162	0.000
Depression							8, 785**	0.018
measurement 1	206	35.35	22.78	288	32.25	22.27	0, 027	0.000
measurement 2	206	30.34	21.98	288	31.88	21.88	1, 676	0.003

COPSOQ – *Copenhagen Psychosocial Questionnaire*.

Psychosocial variables measured with “interaction = type × measurement”, measurement 1 –“between person,” measurement 2 –“within person.”

* p < 0.05;

** p < 0.01;

*** p < 0.001.

a Range 0–100.

b Number of valid responses.

The comparison of psychosocial working conditions and mental well-being concerned the above-mentioned. groups of employees in 2021 and 2022. The analysis was carried out using analysis of variance in a mixed design. The level of psychosocial working conditions was compared in groups of remote and stationary employees participating in the first and second measurement. The results of the ANCOVA regarding the interaction effect of work type and measurement year, as well as the main effects, i.e., the within-person and between-person effects based on the average scores of the 2 measurements, are presented in Table 3. In the case of a significant interaction result, within-person differences were analyzed by group, and the results are shown in Table 3.

**Table 3.** *Post hoc* analysis results for significant ANCOVA interaction effects in remote workers (N = 206) and workers working in the offices (N = 288), nationwide longitudinal study, Poland, 2019–2020

Variable	t	p_Bonferroni_	Cohen's d
Emotional demands			
remote group	2.291	0.022	0.154
stationary group	0.979	1.000	0.013
Work-life conflict			
remote group	2.885	0.025	0.170
stationary group	0.081	0.999	0.002
Burnout			
remote group	3.0808	0.013	0.185
stationary group	0.268	1.000	0.014
Psychological strain			
remote group	2.682	0.045	0.157
stationary group	–0.277	1.000	0.014
Depression			
remote group	4.196	<0.001	0.224
stationary group	0.352	1.000	0.017

**Figure 1.**
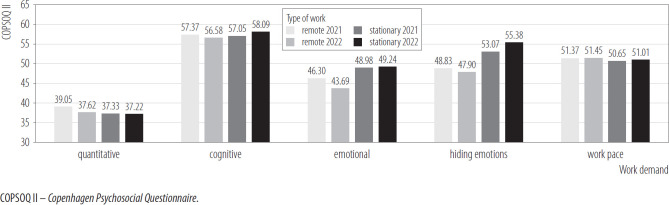
Work requirements in groups of employees working remotely (N = 206) and stationary (N = 288) in 2 measurements, nationwide longitudinal study, Poland, 2019–2020

Figure 1 shows the differences in terms of 5 types of job requirements, in the analyzed groups and measurements. The interaction effects of year and type of work in the mixed design ANCOVA turned out to be statistically insignificant for quantitative demands, cognitive demands, and work pace. Both the between-person and within-person main effects were statistically insignificant for these dependent variables. In the case of requirements to hide emotions the interaction effect was statistically insignificant. However, the between-subject effect out to be statistically significant. Stationary workers exhibited a higher level of requirements to hide emotions than remote workers. In the case of emotional demands, the result of the interaction effect was statistically significant. The remote worker's group scored significantly lower in emotional demands in the second measurement. In contrast, no significant difference was observed in the stationary worker's group. The between-person effect was also significant, with stationary workers scoring higher in emotional demands compared to remote workers, but this effect was small.

**Figure 2.**
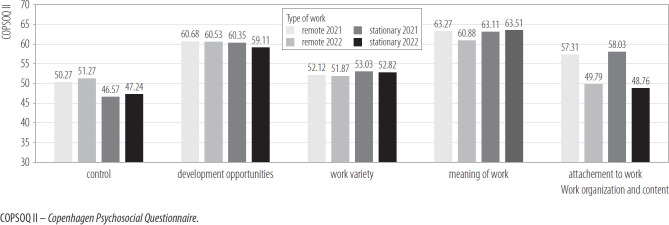
Organization and content of work in groups of employees working remotely (N = 206) and stationary (N = 288) in 2 measurements, nationwide longitudinal study, Poland, 2019–2020

Figure 2 shows the difference between measurements in the remoted and stationary worker. The interaction effects of the mixed model ANCOVA were statistically insignificant in relation to all components, such as sense of influence, development opportunities, work variety, meaning of work, and attachment to the workplace. However, in the case of sense of influence, the test of between-subjects effects occurred statistically. This result indicates that remote workers have more influence over their work than stationary workers have, but this effect was small. In turn, in the case of Attachment to the workplace, a large within-person effect was observed. Both groups of employees in the second measurement assessed their attachment to work lower than in the first measurement. For the remaining variables, all simple main effects were insignificant.

**Figure 3.**
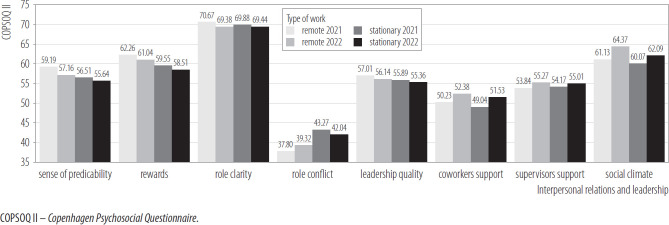
Interpersonal relations and leadership in groups of employees working remotely (N = 206) and on-site (N = 288) in 2 measurements, nationwide longitudinal study, Poland, 2019–2020

The interaction effects of the mixed model ANCOVA turned out to be statistically insignificant in relation to all variables in the area of interpersonal relations, such as sense of predictability, rewards, role clarity, role conflict, leadership quality, support from superiors, support from colleagues, social climate between colleagues. However, in the case of role conflict, the result of the test of between-subject effects turned out to be statistically significant. This result indicates that stationary employees experienced higher role conflict than remote employees, but this effect was small. For the remaining variables presented in Figure 3, the simple main effects were insignificant.

**Figure 4.**
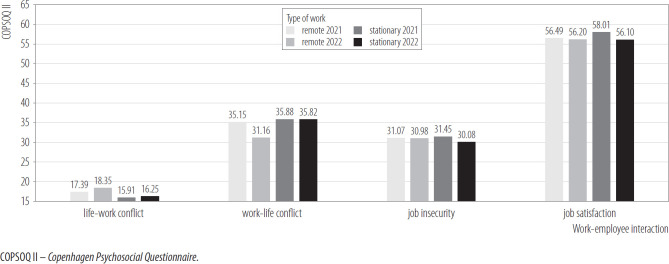
Work-employee interactions in remote (N = 206) and on-site (N = 288) workers in 2 measurements, nationwide longitudinal study, Poland, 2019–2020

Figure 4 presents the differences in the area of humanwork interaction.

The interaction effects of the mixed model ANCOVA in the mixed design turned out to be statistically insignificant in terms of such variables as life-work conflict, job insecurity, job satisfaction. in the case of work-personal life conflict, a significant interaction effect was revealed.

After 1 year, a significant decrease in the level of work-life conflict was observed in the remote workers group. This change was statistically insignificant in the stationary workers group.

**Figure 5.**
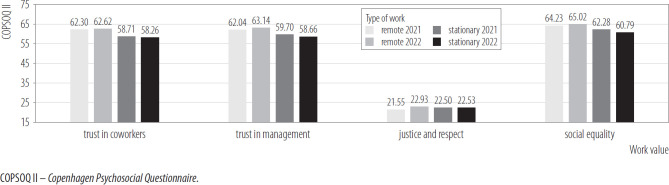
Work values in remote (N = 206) and on-site (N = 288) workers in 2 measurements, nationwide longitudinal study, Poland, 2019–2020

Figure 5 shows the differences in terms of the 4 values at work. The results of the interaction effect were found to be statistically insignificant for all types of work values tested, such as trust between co-workers, trust in management, fairness and respect, and social equality. In the case of trust between co-workers, the result of the between-subject effects test proved statistically significant. This result indicates that remote workers felt higher trust in co-workers compared to stationary workers, but the effect was small. No other significant main effects were noted for the variables presented in Figure 5.

**Figure 6.**
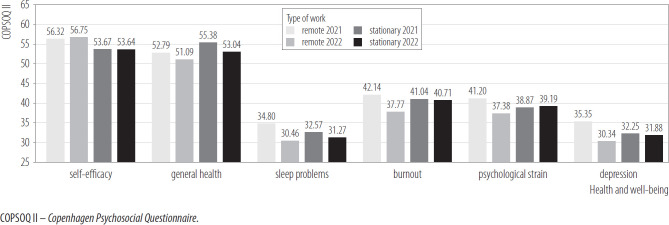
Health and well-being in remote (N = 206) and on-site (N = 288) workers in 2 measurements, nationwide longitudinal study, Poland, 2019–2020

Figure 6 shows the differences in health and well-being indicators. The results of the interaction effect in mixed model ANCOVA proved significant for the variables burnout, psychological strain, and depresson. After 1 year, the remote workers group reported lower levels of psychological strain, burnout, and depression. In contrast, these differences in the on-site workers group were insignificant. The interaction effects for the remaining COPSOQ II variables related to health and well-being were statistically insignificant and also all simple main effects were insignificant.

## DISCUSSION

The results of the surveys of those working remotely and those working on the employer's premises in terms of psychosocial working conditions show that the differences between these groups of people are small. However, several significant effects that did occur indicate that remote workers rated their psychosocial work environment better compared to on-site workers. The latter employees found their work to be more emotionally taxing, requiring them to hide their emotions to a greater extent than remote workers. This result is understandable due to the limited contact with others in remote work. Also, data from other studies clearly indicate that difficult relationships with others (clients, patients, students) are one of the most serious stressors in the workplace [19,20]. Remote working therefore effectively reduces exposure to this stressor. It can be assumed that even the form of this contact (through a communicator) is a factor in alleviating the emotional tension that accompanies a face-to-face meeting. The results of other studies support this conclusion [21]. For many people with social phobia, and even those who experience difficulties in interpersonal relationships, this form of contact with other people is recommended to alleviate the tension that accompanies situations that require personal contact with another person, all the more so if it is a difficult colleague, client or patient.

The higher sense of influence at work in those who work remotely compared to those who work on the employer's premises observed in this research is also fully understandable. This is because remote workers can decide more on the pace of their work, as well as its distribution, and better adapt it to their individual needs, such as the need for rest (breaks) or the need to deal with private matters.

Since a high or forced pace of work is a commonly indicated occupational stressor [20,22,23], remote working could therefore be an excellent prevention of this stressor [24]. Results from other studies indicate that the sense of control is also significantly higher in people who voluntarily work remotely than in those who had no influence on the form of work [25].

With regard to work-life conflict in remote and stationary workers, 2 interesting results were noted. Firstly, in both study groups, the level of work-life conflict was higher than personal-life conflict. This means that work is an extremely important part of a person's life and has a significant impact on its other aspects. Based on this result, it can furthermore be assumed that the respondents were concerned that their personal life did not, however, have an impact on work. In most of the existing studies on this topic in the literature, a result indicating an increase in work-life conflict was obtained in people who worked remotely during the COVID-19 pandemic [26–29]. However, in this study, after 1 year, a significant decrease in this conflict was observed precisely in those who work remotely compared to those who work stationary. This indicates that, in the long term, working remotely maintains this balance better than working at the employer's premises. This conclusion is supported by the results of qualitative research indicating that if remote workers “develop” effective methods of combining work and family care roles over time, the “work-family/private life” conflict decreases significantly [30].

Also, meta-analyses of studies on this topic indicate a similar result to the one obtained in this study: remote working is associated with lower work-life conflict, while the occurrence of significant life-work conflict is not revealed in people working remotely [31].

Data from this study further indicate that remote workers declared higher trust in colleagues than stationary workers. This effect may be explained by the fact that working remotely increases the need, or even the necessity, to interact effectively with others and to obtain possible help from colleagues. Thus, it may enhance trust in those from whom such help is expected [32,33]. During the COVID-19 pandemic, Chong et al. [34] revealed that individuals who were suddenly forced to remotely perform tasks requiring close collaboration felt significantly less work fatigue when they received the necessary support from colleagues and supervisors than when this support was low.

Interesting results were obtained with regard to psychological wellbeing, which (in intergroup effects) did not significantly differ between remote and traditional workers. However, the within-group effect was significant, and in some cases highly significant. Indeed, it turned out that the passage of time had a more positive effect on the wellbeing of those working remotely than on those who worked on the employer's premises. For example, all subjects declared lower levels of burnout in the second measurement compared to the first measurement, i.e., remote workers appeared to be significantly less professionally burned out after 1 year than those who were traditionally employed.

Also with regard to depression, a similar effect was observed. Across the study group, the level of this variable decreased in the second measurement compared to the first measurement, but the difference was significantly greater for remote workers than for stationary workers. Similar results with regard to depression levels were obtained by Henke et al. [35] in a similar longitudinal study performed in a U.S. population.

The effect concerning psychological tension was even more pronounced, as in the second measurement, the level of psychological tension in remote workers decreased significantly, while in the case of stationary workers, a significant increase in the level of this variable was observed. The differences in psychological well-being between the 2 groups of employees observed in this study, indicate that remote working may have been a new phenomenon for Polish employees to which they had to adapt. However, in the long term, this form of work may be more beneficial to the psychological well-being of employees than working at the employer's premises. This effect is confirmed by the results of others [36–38], especially longitudinal studies [39]. This is one of the reasons why this type of study yields more reliable data than those from crosssectional studies.

In the study, the timing of the study may also have played an important role. The first measurement was taken when the COVID-19 pandemic was still ongoing, there were greater restrictions on social life and, presumably, greater anxiety about one's own health. The second measurement took place at a time when the most severe restrictions had been lifted and the state of the epidemic was planned to be lifted, which may have significantly improved the overall psychological wellbeing of Poles.

## CONCLUSIONS

Summarizing the results of the study on the psychosocial aspects of remote working, it can be concluded that remote workers rated some psychosocial conditions of their work better than those working onsite, including the level of emotional demands of the job, the demands of harboring emotions, the sense of influence at work, role conflict and also trust in colleagues. Remote working also had a better impact on the psychological wellbeing of remote workers compared to those working at the employer's premises, particularly in relation to psychological strain, depression and burnout in the long term. These results suggest that it is therefore a desirable form of work from a mental health perspective for employees whose work allows it. However, the authors of existing meta-analyses of studies on this topic emphasize that the impact of working from home (remotely) on an employee's health can vary and that systems need to be developed to optimize this impact [9,40,41]. These include, in particular, technical and organizational support for remote workers, building support networks between workers, offering training to managers on how to manage remote teams, and employee participation in organizational management.
